# Exposure to Allergen Causes Changes in NTS Neural Activities after Intratracheal Capsaicin Application, in Endocannabinoid Levels and in the Glia Morphology of NTS

**DOI:** 10.1155/2015/980983

**Published:** 2015-03-19

**Authors:** Giuseppe Spaziano, Livio Luongo, Francesca Guida, Stefania Petrosino, Maria Matteis, Enza Palazzo, Nikol Sullo, Vito de Novellis, Vincenzo Di Marzo, Francesco Rossi, Sabatino Maione, Bruno D'Agostino

**Affiliations:** ^1^Endocannabinoid Research Group, Section of Pharmacology, “L. Donatelli,” Department of Experimental Medicine, School of Medicine, Second University of Naples, Via Costantinopoli 16, 80138 Naples, Italy; ^2^Endocannabinoid Research Group, Institute of Biomolecular Chemistry, Consiglio Nazionale delle Ricerche, Pozzuoli, 80078 Naples, Italy; ^3^Department of Anaesthesiology, Surgery and Emergency, Second University of Naples, Piazza Luigi Miraglia 2, 80138 Naples, Italy

## Abstract

Allergen exposure may induce changes in the brainstem secondary neurons, with neural sensitization of the nucleus solitary tract (NTS), which in turn can be considered one of the causes of the airway hyperresponsiveness, a characteristic feature of asthma. We evaluated neurofunctional, morphological, and biochemical changes in the NTS of naive or sensitized rats. To evaluate the cell firing activity of NTS, in vivo electrophysiological experiments were performed before and after capsaicin challenge in sensitized or naive rats. Immunohistochemical studies, endocannabinoid, and palmitoylethanolamide quantification in the NTS were also performed. This study provides evidence that allergen sensitization in the NTS induced: (1) increase in the neural firing response to intratracheal capsaicin application, (2) increase of endocannabinoid anandamide and palmitoylethanolamide, a reduction of 2-arachidonoylglycerol levels in the NTS, (3) glial cell activation, and (4) prevention by a Group III metabotropic glutamate receptor activation of neural firing response to intratracheal application of capsaicin in both naïve and sensitized rats. Therefore, normalization of ovalbumin-induced NTS neural sensitization could open up the prospect of new treatments based on the recovery of specific brain nuclei function and for extensive studies on acute or long-term efficacy of selective mGlu ligand, in models of bronchial hyperreactivity.

## 1. Introduction

Bronchial hyperresponsiveness (BHR), a characteristic feature of asthma, may be exacerbated by various local inflammatory mediators released by repeated exposures to allergen [1, 2]. Over the last few years, it has been shown that several inflammation-generated mediators induce long-term functional modifications of the sensory airway neural pathways in rodent and primate models of asthma: neuroplastic changes in the peripheral airway afferent nerves as well as in the brainstem secondary neurons and/or motor vagus output neurons have been demonstrated [3]. The direct consequence of neuroplasticity in the brainstem nucleus of solitary tract (NTS) or the dorsal motor nucleus of vagus is mainly represented by neural sensitization which in turn may be considered one of the causes of the BHR to various bronchoconstrictor stimuli [4].

Over the last decade, evidence has accumulated on the complex biomolecular mechanisms related to neural sensitization and plasticity, which are critical for a variety of phenotypic changes in neuron activities [5]. These functional changes are considered to be at the basis both of several physiological events such as memory and learning [5, 6] and of many pathological conditions, such as chronic pain syndromes [7]. Indeed, enduring neuropathic or inflammatory pain is a well-characterized pathophysiological condition in which a direct parallel between persistent exposure to excitatory/inflammatory neurotransmitters and the increased excitability of spinal post-synaptic neurons has been clearly shown [8–11]. Many studies have proposed an analogy between airway hyperresponsiveness and hyperalgesia. Considering that the endovanilloid oleoylethanolamide excites sensory vagal neurons via TRPV1 receptors [12] and that BHR mediated by several stimuli [13, 14] is abolished following chronic treatment with capsaicin; sensory nerves can represent a common pathway by which many stimuli can induce BHR. These studies are consistent with the hypothesis that “sensitization” of airway sensory nerves may contribute toward this phenomenon [15].

Further confirmation of a similarity between the neural adaptive mechanisms for airway neural sensitization and the establishment chronic pain is the fact that both phenomena share the same neurotransmitters and neuromodulators (i.e., glutamate, SP, GABA, endocannabinoids, etc.) at both peripheral (lung and trachea) and brainstem levels [3, 16, 17]. In particular, the endocannabinoids anandamide and 2-arachidonoylglycerol (2-AG) and their main cannabinoid CB1 and CB2 receptors have been identified in the NTS [18], where another molecular target of anandamide, the transient receptor potential vanilloid type-1 (TRPV1) channel, is also abundantly coexpressed with CB1 receptors [17].

Moreover, the anandamide congener palmitoylethanolamide (PEA), which activates peroxisome proliferator-activated receptor (PPAR)-*α*, can also enhance anandamide actions at CB1 and TRPV1 receptors [19]. Importantly, NTS TRPV1 channel stimulation by capsaicin was shown to induce the cough reflex in the guinea pig [20], whereas CB1 receptors in this nucleus seem to be more involved in the control of emesis, oesophageal sphincter relaxation, and baroreflex-evoked sympathoinhibition [17, 18, 21, 22].

Based on these considerations, we have evaluated some functional, morphological, and biochemical changes occurring in the NTS following airway sensory nerve activation in naive and ovalbumin-sensitized rats. In particular, we evaluated (i) the responsiveness of the intrinsic NTS neurons by intratracheal application of capsaicin; (ii) the levels of the two major endocannabinoids, anandamide, and 2-AG and of the cannabinoid receptor-inactive PEA; (iii) the morphological changes in NTS microglia and astroglia. Group III metabotropic glutamate receptors include mGlu4, mGlu6, mGlu7, and mGlu8 mainly located on presynaptic terminals where they modulate neurotransmitter release. L-(+)-2-amino-4-phosphonobutyric acid (L-AP4), L-serine-O-phosphate (L-SOP), and (1S, 2R)-1-amino-phosphonomethylcyclopropane carboxylic acid (1S, 2R)-APCP are broad spectrum agonists whereas L-AP4 and L-SOP *α*-methyl analogs, (S)-*α*-methyl-2-amino-4-phosphonobutanoic acid (MAP4), and (RS)-*α*-methylserine-O-phosphate (MSOP) behave as antagonists. Since group III mGlu receptor modulate local neurokinins and glutamate releases [23], we also analyzed their roles in NTS neuron activities before and after capsaicin-induced C-fibers afferent nerve activation.

## 2. Materials and Methods

### 2.1. Animals

Male Norway brown rats (250–300 g) were housed 3 per cage under controlled illumination (12 : 12 h light : dark cycle; light on 06.00 h) and environmental conditions (ambient temperature 20–22°C, humidity 55–60%) for at least 1 week before the commencement of experiments. Rat chow and tap water were available* ad libitum*. The experimental procedures were approved by the Animal Ethics Committee of the Second University of Naples. Animal care was in compliance with Italian (D.L. 116/92) and EEC (O.J. of E.C. L358/1 18/12/86) regulations on the protection of laboratory animals. All efforts were made to minimise animal suffering and to reduce the number of animals used.

### 2.2. Sensitization

The rats were sensitized by a subcutaneous (sc) injection of 0,66 mL of a suspension of 1 mg OVA plus 300 mg of aluminium hydroxide in 0,9% NaCl solution (saline) [24]. Naïve rats received saline only. This was considered Day 1 of sensitization. Seven days after sensitization, the animals were boosted subcutaneously (sc) with an identical injection of ova suspension. Twenty one days after the initial injection, animals were challenged with 5% aerosolized OVA. OVA was aerosolized for 5 min using an ultrasonic nebuliser and nebuliser control unit (Buxco Electronics). On the day 22, 24 hours after the OVA challenge, bronchopulmonary function was performed. Control animals were challenged with 0.9% saline solution. To evaluate the successful of OVA sensitization, five rats of each group (sensitized and naive) were used to assess airway responsiveness. Animal were anaesthetized by an i.p. injection of urethane (1.3 g/kg, i.p.) and lung function was assessed 30 min later. The anaesthetized rats were exposed to sterile saline for 2 min and lung functions were recorded. Airway responsiveness (*R*_*L*_) was measured following aerosol administration of double concentrations of metacholine for 30 s and measurements of respiratory parameters were taken every minute for 5 min. Peak value of *R*_*L*_ was measured after each concentration and the challenge was stopped at 128 mg/mL metacholine. We have measured the concentrations of metacholine inducing 200% increase of *R*_*L*_ over the initial baseline (EC_200_ *R*_*L*_).

### 2.3. Experimental Protocol

Groups of 5 animals per treatment were used with each animal being used for one treatment only.

A group of naive rats was implanted with guide cannulae and received an intracerebral microinjection of 2 microliters of ACSF and served as a control of the intracerebral drug microinjection.

For the in vivo extracellular recording, naive and sensitized rats were grouped as follows.

- Groups of naive or sensitized rats received intracerebral administration of L-AP4 (2 and 4 nmol/rat) alone or L-AP4 (4 nmol/rat) in combination with MSOP (100 nmol/rat). When L-AP4 was administered in combination with MSOP, the latter was centrally delivered 5 min before the administration of L-AP4.
- Groups of naive or sensitized rats received intracerebral administration of MSOP (100 and 300 nmol).

All groups received intratracheal capsaicin challenge (300 pg in 20 *μ*L) or respective vehicle. In a separate set of experiments, groups of sensitized and naive rats were killed with a lethal dose of pentobarbital and decapitated for assay of endocannabinoid content and for the immunohistochemistry analysis in the NTS area. The doses were chosen according to previous data [25].

### 2.4. Preliminary Surgical Preparations

Each rat was anaesthetized with an i.p. injection of pentobarbital (50 mg/kg). A catheter was introduced into the jugular vein for administering saline or for the continuous infusion of propofol (5–10 mg/kg/h) to maintain a constant anaesthesia. Trachea was cannulated below the larynx, and a tiny catheter was also connected to a side-part of that cannula to allow intratracheal vehicle or capsaicin (300 pg in 20 *μ*L) application. The cervical vagus nerve ipsilateral to the recording site was isolated (mainly the right side) for the placement of the stimulating electrode. In order to perform administrations of drug or respective vehicle (artificial cerebrospinal fluid, ACSF, composition in mM: KCl 2.5; NaCl 125; MgCl_2_ 1.18; CaCl_2_ 1.26) into the cerebral lateral ventricle, a 23-gauge, 12 mm-long stainless steel guide cannula was stereotaxically lowered until its tip was 1.5 mm above the ventricle by applying coordinates from the atlas of Paxinos and Watson [26] (A: 0.92 mm and L: 1.5 mm from bregma, V: 2.9 mm below the dura).

These coordinates were chosen in order to have enough space to allow stereotaxic manipulation for the positioning of both the guide cannula for drug microinjection and of the tungsten electrode for the in vivo NTS cell recording. The guide cannula was anchored with dental cement to a stainless steel screw in the skull. We used a David Kopf stereotaxic apparatus (David Kopf Instruments, Tujunga, CA, USA) with the animal positioned on a homeothermic temperature control blanket (Harvard Apparatus Limited, Edenbridge, Kent). The guide cannula for drug microinjection was implanted on the same day as the electrophysiological recording. Direct intracerebral administration of drugs or respective vehicle was conducted with a stainless steel cannula connected by a polyethylene tube to a SGE 1-microlitre 26-gauge syringe, inserted through the guide cannula and extended 1.5 mm beyond the tip of the guide cannula to reach the cerebral ventricle. Volumes of 2 *μ*L drug solutions or vehicle were injected into the ventricle over a period of 60 s and the injection cannula gently removed 2 min later.

### 2.5. NTS Extracellular Recording

After implantation of the guide cannula into the cerebral ventricle, a tungsten microelectrode was stereotaxically [26] lowered through a small craniotomy to record the activity of the airway-related NTS neurons before and after intratracheal application of capsaicin. These neurons were identified by stimulating the vagus nerve (200–600 *μ*A, 0.5–0.8 ms pulses) at 1 Hz during the slow (1 *μ*m s) electrode lowering within the NTS [14, 27]. Extracellular single-unit recordings were made in the NTS with glass insulated tungsten filament electrodes (3–5 MΩ) (FHC Frederick Haer & Co., ME, USA) using the following stereotaxic coordinates: 3–3.6 mm caudal to lambda, 1–1.5 mm lateral, and 7.7–8.1 mm depth from the surface of the brain [26]. The recorded signals were amplified and displayed on analog and digital storage oscilloscope to ensure that the unit under study was unambiguously discriminated throughout the experiment. Signals were also fed into a window discriminator, whose output was processed by an interface (CED 1401) (Cambridge Electronic Design Ltd., UK) connected to a Pentium III PC. Spike2 software (CED, version 4) was used to create peristimulus rate histograms online and to store and analyse digital records of single-unit activity offline. Configuration, shape, and height of the recorded action potentials were monitored and recorded continuously, using a window discriminator and Spike2 software for on-line and off-line analysis. Once an NTS unit was identified from its background and tracheal/vagus stimulation activity, we optimised spike size before all treatments. This study only included neurons whose spike configuration remained constant and could clearly be discriminated from activity in the background throughout the experiment, indicating that the activity from one neuron only and from that same neuron was measured. Only one neuron was recorded in each rat and the recording RVM site was marked with a 20 *μ*A DC current for 20 s.

### 2.6. Endocannabinoid Extraction and Quantification

#### 2.6.1. Analysis of Endocannabinoid Contents

Anaesthetized rats were decapitated and their brains were rapidly removed and immersed in oxygenated ice-cold artificial cerebrospinal fluid. A block of tissue containing the NTS was cut using a vibrotome (Vibratome 1500, Warner Instruments, CT, USA). A brainstem slice of 2–2.5 mm was cut throughout the medulla containing the NTS region and using the following stereotaxic coordinates: 3–3.6 mm caudal and 1–1.5 mm lateral to lambda [26]; the right and left NTS from the same rat were isolated under microscope (M650, Wild Heerbrugg, Switzerland) and pooled. Tissues were homogenized in 5 vol of chloroform/methanol/Tris HCl 50 mM (2 : 1 : 1) containing 50 pmol of d_8_-anandamide, d_4_-palmitoylethanolamide, and d_5_-2-AG. Deuterated standards were synthesized from d_8_ arachidonic acid and ethanolamine or arachidonic acid and d_5_-glycerol or d_4_-palmitic acid and ethanolamine. Homogenates were centrifuged at 13,000 g for 16 min (4°C), the acqueous phase plus debris were collected and extracted again twice with 1 vol of chloroform. The organic phases from the three extractions were pooled and the organic solvents evaporated in a rotating evaporator. Lyophilized samples were then stored frozen at −80°C under nitrogen atmosphere until analyzed and were resuspended in chloroform/methanol 99 : 1 by vol. The solutions were then purified by open bed chromatography on silica as described by Maione et al. [28]. Fractions eluted with chloroform/methanol 9 : 1 by vol. (containing anandamide, pamitoylethanolamide, and 2 AG) were collected and the excess solvent evaporated with a rotating evaporator, and aliquots analyzed by isotope dilution-liquid chromatography/atmospheric pressure chemical ionisation/mass spectrometry (LC APCI-MS) carried out under conditions described previously [28] and allowing the separations of 2-AG, palmitoylethanolamide, and anandamide. MS detection was carried out in the selected ion monitoring mode using* m/z* values of 356 and 348 (molecular ion+1 for deuterated and undeuterated anandamide), 304.0 and 300.0 (molecular ion+1 for deuterated and undeuterated palmitoylethanolamide), and 384.35 and 379.35 (molecular ion+1 for deuterated and undeuterated 2 AG). The area ratios between signals of deuterated and undeuterated anandamide varied linearly with varying amounts of undeuterated compounds. Anandamide, palmitoylethanolamide, and 2 AG levels in unknown samples were therefore calculated on the basis of their area ratios with the internal deuterated standard signal areas.

### 2.7. Immunohistochemistry 

Under pentobarbital anaesthesia animals were transcardially perfused with 0.9% saline solution followed by 4% paraformaldehyde in 0.1 M phosphate buffer. The brain was excised, postfixed for 4 hr in the perfusion fixative, cryoprotected for 72 h in 20% sucrose in 0.1 M phosphate buffer, and frozen in O.C.T embedding compound. 20 *μ*m transverse sections were cryostat cut and thaw-mounted onto glass slides. The NTS was identified based on the Paxinos and Watson Atlas coordinates (1986) [26]. Slides were incubated overnight with primary antibody solutions for the microglial cell marker Iba-1 (Rabbit anti-ionized calcium binding adapter molecule 1; 1 : 1000; Wako Chemicals, Germany), the astrocytes marker GFAP (Glial fibrillary acidic protein; 1 : 1000; DAKO, USA). Following incubation sections were washed and incubated for 3 hr with secondary antibody solution (goat anti-rabbit, IgG-conjugated Alexa Fluor 488; 1 : 1000; Molecular Probes, USA). Slides were washed, cover-slipped with Vectashield mounting medium (Vector Laboratories, USA), and visualised under a Zeiss Axioplan 2 fluorescent microscope.

Quantitative assessment was carried out by determining the intensity of positive profiles for each marker within a fixed area of the NTS. A box measuring 10^4^ *μ*m^2^ was placed onto areas of the lateral, central, and medial NTS and the intensity of positive profiles within this area recorded by a AxioVision Rel. 4.6 program. This measurement protocol was carried out on three NTS sections from each animal.

### 2.8. Drugs

Capsaicin (Sigma-Aldrich, Milano Italy) was dissolved in a solution consisting of ethanol and water (6 : 4).

L-2-Amino-4-phosphonobutyric acid and (RS)-*α*-methylserine-o-phosphate were purchased by Tocris Bioscience, Bristol, UK, and dissolved in ACSF.

### 2.9. Statistics

For electrophysiological study, the single extracellular recording (action potentials) was analysed offline from peristimulus rate histograms using Spike2 software (CED, version 4). The neuron responses, before and after capsaicin-induce stimulation or following intracerebroventrolateral vehicle or drug microinjections, were measured and expressed as spikes/sec (Hz). In particular, basal values were obtained by averaging the activities recorded 10 min before drug applications. Data are presented as mean ± standard error (S.E.) of changes in neuron responses (extracellular recordings).

Statistical comparisons of values from different treated groups of rats were made using the two-way analysis of variance (ANOVA) for repeated measures followed by the Tukey/Kramer test for post hoc comparisons. Comparisons between pre- and posttreatment ongoing activity and capsaicin-related cell burst were performed by applying the nonparametric Wilcoxon matched-pairs signed rank test.

Mean values for each group were then compared using Student's* t*-test. *P* < 0.05 was set as the level of statistical significance.

The amounts of endocannabinoids were expressed as picomoles or nanomoles per gram of wet tissue extracted and were compared by ANOVA followed by Bonferroni's test.

## 3. Results 

### 3.1. Airway Responsiveness and Endocannabinoid Levels Measurements of Naive and Sensitized Rats

Baseline absolute value of *R*_*L*_ and *C*_dyn_ was not significantly different between two groups.

**Figure 1:**
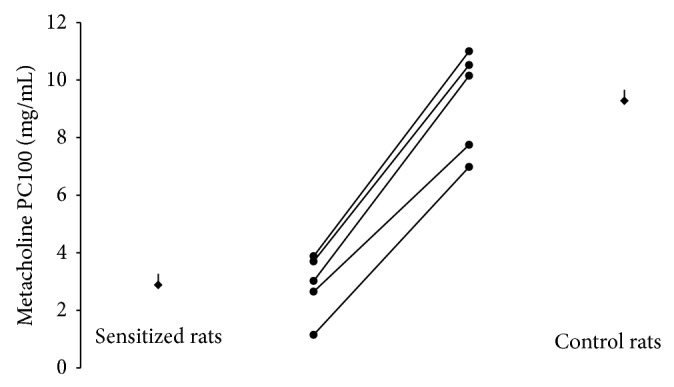
Airway responsiveness to methacholine in ovalbumin (sensitized) or saline treated rats (control) (*n* = 5). ^**^ *P* < 0.01 compared with control group. Circles represent single values and squares represent mean values.

In sensitized rats, OVA aerosol caused an acute bronchoconstriction, with an approximately threefold greater increase in *R*_*L*_ and decrease in *C*_dyn_ respect to an aerosol of saline solution (data not shown). OVA challenge exposure resulted in an increase of airway responsiveness to inhaled histamine, approximately fourfold (*P* < 0.01) when compared with saline challenge (PC_100_: 69 mg/mL; 18 mg/mL saline and ova groups, resp.) (Figure 1). Moreover, sensitization caused an enhancement of the endogenous TRPV1/CB1 “hydrid” agonist, anandamide, and of the endogenous PPAR*α* agonist palmitoylethanolamide levels in the NTS area, whilst lowering the levels of the CB1-selective endocannabinoid 2-AG (Table 1).

### 3.2. The Effect of Intratracheal Capsaicin on Airway-Related NTS Neuron Activities in Naive or Sensitized Rats

**Figure 2:**
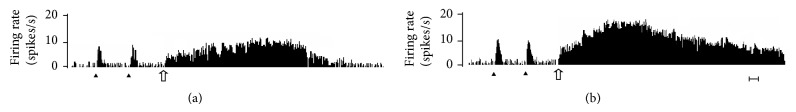
Example of ratemeter records which illustrate the spontaneous activity of NTS neurons before and after capsaicin administration (hollow arrow). (a) control rat, (b) ovalbumin-sensitized rat. A single oscilloscope trace (2 min recording) shows spontaneous activity of a single unit (long black arrow) immediately before and after intratracheal capsaicin application. Small black arrowheads indicate single vagal stimulations. Scale bar = 1 min.

The results are based on airway-related NTS neurons (group size = 5; one cell recorded from each animal per treatment) at a depth of 7.7–8.1 *μ*m from the surface of the brain, the estimated location of the neurons being within the NTS. All recorded neurons showed very little spontaneous activity (they frequently paused completely during 20–50 s) and discharged with a mean frequency of 0.7 ± 0.03 spikes/s. These neurons were identified by an increased burst of activity just after vagus nerve stimulation (Figure 2).

Intratracheal application of capsaicin (300 pg in in 20 *μ*L) induced an increase in the firing activity of the airway-related NTS neurons in naive rats, which was maximal (9.9 ± 0.7 spikes/s) 20 min after the administration of capsaicin (Figure 2(a)). In sensitized rats, intratracheal application of capsaicin induced a higher increase in the firing activities (12.4 ± 0.6 spikes/s) of the airway-related NTS neurons than it did in naive rats. Unlike the naive rats, we did not observe any recovery in the sensitized rats during the observation period (60 min post-capsaicin) (Figure 2(b)).

### 3.3. The Effect of L-AP4 on Capsaicin-Induced Change on the Airway-Related NTS Neuron Activities in Naive and Sensitized Rats

Intracerebroventricular microinjections of L-AP4 (2–4 nmol/rat) did not induce any effect on the basal value of airway-related NTS cells ongoing activities (data not shown).

**Figure 3:**
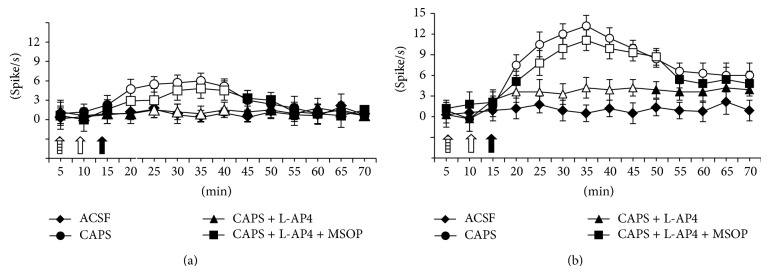
Effect of vehicle (20% DMSO in ACSF) or capsaicin (300 pg in 20 *μ*L) in naïve (a) and sensitized (b) rats. L-AP4 (4 nmol/rat) (hollow arrow) prevented the capsaicin (full arrow) induced increase in the airway-related NTS neuron ongoing activities. This effect of L-AP4 (4 nmol/rat) was prevented by pretreatment with MSOP (100 nmol/rat) (arrow with lines). Each point represents the mean ± SEM of five rats per group. Values statistically (*P* value < 0.05) significant versus the respective control were indicated as open symbols.

The highest doses of L-AP4 (4 nmol/rat) prevented the capsaicin-induced increase in the airway-related NTS neuron activities in both naive and sensitized rats (Figures 3(a) and 3(b)). The effects of L-AP4 (4 nmol/rat) were prevented by pretreatment with MSOP (100 nmol/rat), which* per se *did not significantly change the airway-related NTS neuron (Figures 3(a) and 3(b)).

### 3.4. Immunohistochemistry

**Figure 4:**
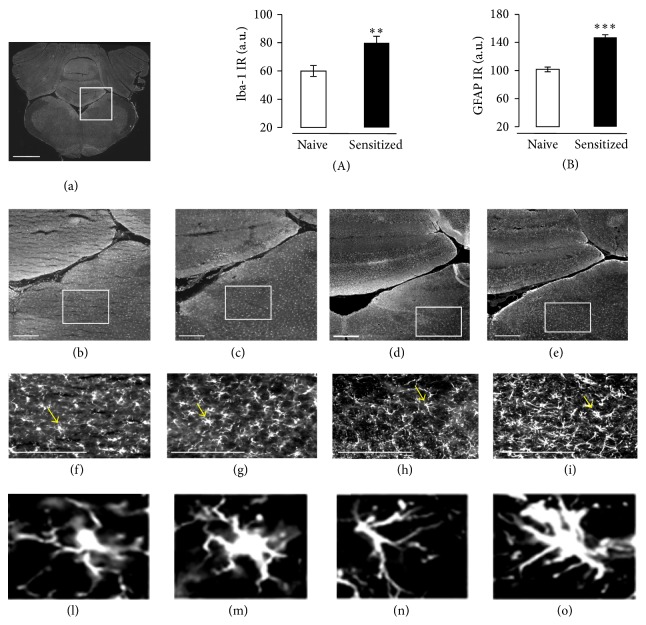
(a) Low magnification of NTS brain area and schematic representation (see Paxinos and Watson, 1986 [26]) of the area beside. (b, f, l) Iba-1 IR in NTS of a naive rat. (c, g, m) Iba-1 IR in NTS of sensitized rat to ovalbumin. (d, h, n) GFAP IR in NTS of a naive rat. (e, i, o) GFAP IR in NTS of sensitized rat. High magnification of Iba-1 + profiles (l, m) and GFAP + profiles (n, o) arrows. Scale bars = 100 *μ*m. (A, B) Quantitative analysis of Iba1 and GFAP staining in NTS reveals significantly increased numbers of Iba-1 and GFAP-positive cells in the NTS after ovalbumin sensitization. Data represented as mean ± SEM, *n* = 3 rats per group. ^**^ *P* < 0.01, ^***^ *P* < 0.001 compared to control group, one-way ANOVA, post hoc Tukey.

Immunoreactivity (IR) for the microglial cell marker Iba-1 was observed in the NTS of control and sensitized animals. In sensitized animals, the increased expression of Iba-1 and specific morphological changes, such as the increased thickness of cell bodies and process retraction, suggest activation of microglia. In particular, quantitative analysis of Iba-1 IR revealed a significant increase in the intensity of Iba-1 positive cells in the NTS of sensitized rats (79.9 ± 4.6 arbitrary units), in comparison to naive animals (58.9 ± 3.8 arbitrary units). As far as the analysis of astrocyte activity is concerned, IR for marker GFAP was evaluated in the NTS of control and sensitized animals. In sensitized animals, we observed an increased expression of GFAP and specific morphological changes, such as increased astrocyte cell body and process thickness, assuming reactive astrogliosis following sensitization to ovalbumin. In particular, GFAP IR quantitative analysis revealed a significant increase in the intensity of GFAP positive cells in the NTS of sensitized rats (147.1 ± 3.8 arbitrary units), in comparison to control animals (101.6 ± 3.3 arbitrary units) (Figure 4).

## 4. Discussion

This study shows that ovalbumin-induced sensitization increases: (1) the NTS neural firing response to intratracheal capsaicin application, (2) the endocannabinoid anandamide level, and (3) astro- and microgliosis in the NTS. Moreover, we also show that the intracerebroventricular application of a Group III metabotropic glutamate receptor agonist prevents the neural firing response to the intratracheal application of capsaicin in both naïve and sensitized rats. The overall hypothesis linking these different findings to the generation of bronchial hyperresponsiveness (BHR) is based on the possibility that peripheral nerve sensitization such as, for example, during persistent inflammation, may induce long-lasting pathophysiological modifications in the NTS neural and glial cell functioning. Indeed, in a similar way to the changes observed in the spinal cord in chronic pain [7, 9], we suggest that, also in this case, a higher discharge of the afferent sensitized neurons may increase the release of excitatory neurotransmitters (i.e., glutamate and CGRP) in the NTS responsible for neurons, astrocytes, and microglia phenotypic modifications [29]. Importantly, pathophysiological conditions like chronic pain or inflammation are associated with alterations in the levels of some on-demand produced endocannabinoid/endovanilloids such as anandamide, PEA, or 12-lipoxygenase products (i.e., 12-HPETE) [30–33].

Consistently with this possibility, the increase in the NTS neural firing response to intratracheal application of capsaicin in sensitized animals has led us to believe that these cells might be hyperactive airway-activated NTS neurons [34]. Considering that in this study intratracheal capsaicin induced a higher and long-lasting firing discharge of the airway-related NTS neurons, one might speculate that similarly to previous findings [35–37], chronic allergen challenge can lead to persistent inflammation and activation of afferent vagal fibers modulating the activity of the airway-activated NTS neurons. Indeed, persistent stimulation on the small-diameter nerve fibers (i.e., C-fibers and A*δ*-fibers) by several direct and indirect acting chemical mediators in the lung may be responsible for afferent neuron sensitization and for phenotypic modifications in NTS cell functioning [29]. In line with this study, NTS neural sensitization in slices of asthmatic primates was shown by Chen and colleagues [38], and it may be possible that the electrophysiological effects observed here could also be related to the increased activity of microglia and astrocytes in the NTS that, in turn, can alter the synaptic plasticity. Accordingly, with the idea that glia plays critical role in determining or sensing neuronal well-being and is capable of shaping neural activities either in healthy or in several pathological brain conditions [39–41], our current findings demonstrate the occurrence of gliosis in the NTS of albumin-sensitized rats. However, it is also intriguing that in many cases gliosis has two faces, protective or deleterious, and understanding of the rules governing this duality is still in its initial stages [42]. Nevertheless, there is evidence to suggest that neurons and glia mutually affect their functioning through complex, not fully explored mechanisms [39, 43] generating alterations in the levels of excitatory (i.e., glutamate and CGRP) and inhibitory (i.e., GABA and endocannabinoids) neurotransmitters. In particular, the recently identified endovanilloids/endocannabinoids are capable of glia activation/differentiation and play roles in neurodegenerative disorders accompanied by microglial activation [44–46]. Regarding endocannabinoid involvement in the modulation of the NTS neural activities, it has been shown that, by acting on presynaptic cannabinoid CB1 receptors, they inhibit both excitatory and inhibitory signalling in the NTS [47]. In contrast, by activating TRPV1 receptors, the endocannabinoid anandamide stimulates glutamatergic signalling [48], with subsequent (1) stimulation of GABA release in this nucleus and a subsequent decrease in NTS neuronal firing [48]; and/or (2) stimulation of output neurons; two effects that would reduce and increase bronchoconstriction (in the latter case via reflex output disinhibition and increased bronchoconstrictive reflexes), respectively. However, the effect of CB1 receptor activation has so far mostly been related to the control of emesis, lower visceral functions, and blood pressure [17, 18, 21, 22].

In order to preliminarily evaluate a role of the endocannabinoid system at NTS level, we measured the content of the two more representative endocannabinoids, anandamide, and 2-AG, as well as of the PPAR-*α* endogenous agonist that also enhances anandamide actions at CB1 and TRPV1 receptors, palmitoylethanolamide (PEA). Here, we show that airway sensitization is accompanied by a statistically significant enhancement of anandamide and PEA, whereas it induced a reduction in 2-AG levels in the NTS. Although endocannabinoids like anandamide might inhibit synaptic transmission via CB1 receptors in the NTS [47], we found that the overall endocannabinoid tone in this nucleus might remain unchanged or have even decreased following airway sensitisation, because of the opposite changes of anandamide and 2-AG levels and the fact that basal 2-AG levels are higher than AEA levels. Indeed, a reduction rather than an increase in cannabinoid receptor activity would be more in line with the increased microglial and glial cell density observed here in the NTS following ovalbumin sensitisation, since (1) CB1 receptor activation was recently shown to inhibit gliosis induced by a *β*-amyloid peptide [49]; (2) both CB1 and CB2 receptor activations were found to inhibit the release of proliferation- and motility-inducing cytokines from astrocytes [50]; and (3) CB2 receptor agonists inhibit microglial cell activation in animal models of neuroinflammatory disorders [51, 52]. On the other hand, the elevated levels of anandamide and PEA in the NTS might underlie the increased microglial density observed in this nucleus following ovalbumin sensitisation, since these two neurotransmitters synergistically stimulate microglial migration via non-CB1 non-CB2 receptors [53]. Conversely, since microglial cells produce more PEA and anandamide than 2-AG [54], the observed changes in NTS levels of these compounds might be due to the elevated active microglia found in sensitised rats. It is worth noting that opposing regulation on anandamide and 2-AG levels is not unprecedented in literature, and a recent finding indicates that, by activating TRPV1, anandamide might in fact reduce 2-AG biosynthesis in the striatum [55, 56].

The above observations suggest that anandamide may preferentially activate TRPV1 in the NTS than cannabinoid receptors, as has been observed in the periaqueductal grey following administration of intermediate doses of an inhibitor of anandamide enzymatic hydrolysis [28]. In the presence of (1) concomitantly elevated levels of PEA, which potentiates anandamide effects at TRPV1 [57], and is unlikely to act via PPAR-*α* (which has never been described as being expressed in the NTS), and (2) reduced CB1 tone, which disinhibits TRPV1 activity [56], anandamide activity at these channels might be enhanced further. The subsequent enhancement of glutamatergic signalling would either stimulate NTS output neuron activity, thus contributing to sensitisation-induced airway hyperresponsiveness. Alternatively, it could enhance GABAergic signalling and hence counteract NTS neuronal firing [48] and bronchoconstriction, representing an adaptive response to sensitization. The former possibility is supported by our finding that the blockade of glutamatergic signalling by Group III metabotropic glutamate receptors activation reduces capsaicin-induced elevation of NTS neuronal activity. However, since prolonged activation of TRPV1 can also cause its desensitisation, it is possible that ovalbumin-induced elevation of anandamide, by desensitising TRPV1, acts in a similar way to Group III mGlu receptor activation.

Indeed, vagal brainstem circuits seem to be organized in such a way that Group II subtype receptors (mGlu2 and mGlu3) are expressed on GABAergic and glutamatergic intrinsic NTS neurons, whereas Group III mGlu receptors seem to be mainly expressed on glutamatergic nerve terminals impinging on output preganglionic neurons [23]. While NMDA and AMPA/Kainate receptor contribute to the excitatory inputs and in the activity-dependent plastic changes of NTS during airway hyperreactivity [58, 59]. Group III mGlu receptors have been shown to significantly contribute to the depression of autonomic signal transmission by attenuating the presynaptic release of glutamate and neurokinins [38]. In this study, the intracerebroventricular administration of L-AP4, the relative selective Group III metabotropic glutamate receptor agonist, prevented the airway-related NTS neuron discharges induced by intratracheal capsaicin in naïve and sensitized rats and confirming their modulatory role in glutamate release. Moreover, it is worth noting that the same dose of L-AP4 prevented capsaicin-induced NTS cell discharges in both naïve and sensitized rats. This observation allows us to speculate that the occurrence of desensitization mechanisms for Group III mGlu receptors might be excluded in the sensitization model applied here. If this may represent an additional advantage in the potential management of bronchial hyperreactivity with selective mGlu receptor ligands, it is obvious that extensive studies are needed in order to examine their possible systemic use and efficacy in different in vivo models of bronchial hyperresponsiveness or asthma in more detail.

## 5. Conclusions

In conclusion, we found that the allergen sensitization in the NTS induced (1) an increase in the neural firing response to intratracheal capsaicin application, (2) an endocannabinoid anandamide increase, and (3) glial cell activation. Although the pathophysiological significance of these different findings remains to be assessed, they could however be relevant to the altered NTS neurotransmitter and cellular morphofunctional changes, which in turn might be collectively involved in the long-lasting NTS cell phenotypic modifications. The overall hypothesis is that the different findings are not independent events, but are direct consequence of the peripheral nerve sensitization which is in turn capable of inducing long-lasting airway-related NTS neural sensitization and hence bronchial hyperresponsiveness. Interestingly, it was also found that acute intracerebroventricular application of a Group III metabotropic glutamate receptor agonist prevented a neural firing response to intratracheal application of capsaicin in both naïve and sensitized rats. Normalization of ovalbumin-induced NTS neural sensitization opens up the prospect of new treatments based on the recovery of specific brain nuclei function and for extensive studies to examine the acute or long-term efficacy of selective mGlu ligand in specific models of bronchial hyperreactivity in greater detail.

## Figures and Tables

**Table 1:** Endocannabinoid levels in the NTS area.

	NTS	
	Control	Sensitized
Anandamide (pmol/g)	32.0 ± 3.0	49.9 ± 3.1^*^
2-AG (nmol/g)	7.4 ± 0.1	6.5 ± 0.1^*^
Palmitoylethanolamide (pmol/g)	961.4 ± 16.4	1187.8 ± 43.4^*^

Data are means ± SEM of *n* = 4 separate experiments. ^*^ *P* < 0.05 as assessed by ANOVA followed by Bonferroni's test.

## Abbreviations

NTS: Nucleus solitary tract
OVA: Ovalbumin
BHR: Bronchial hyperresponsiveness
*R*_*L*_: Lung resistance
*C*_dyn_: Dynamic compliance
TRPV1: Transient receptor potential vanilloid type 1.
